# A direct real-time polymerase chain reaction assay for rapid high-throughput detection of highly pathogenic North American porcine reproductive and respiratory syndrome virus in China without RNA purification

**DOI:** 10.1186/2049-1891-5-45

**Published:** 2014-10-02

**Authors:** Kang Kang, Keli Yang, Jiasheng Zhong, Yongxiang Tian, Limin Zhang, Jianxin Zhai, Li Zhang, Changxu Song, Christine Yuan Gou, Jun Luo, Deming Gou

**Affiliations:** College of Animal Science and Technology, Northwest A&F University, Yangling, Shaanxi 712100 China; College of Life Sciences, Shenzhen Key Laboratory of Microbial Genetic Engineering, Shenzhen University, Shenzhen, 518060 China; Hubei Key Laboratory of Animal Embryo and Molecular Breeding, Institute of Animal Husbandry and Veterinary, Hubei Academy of Agricultural Sciences, Wuhan, 430064 China; Shenzhen Ao Dong Inspection and Testing Technology Co,. Ltd, Shenzhen, 518000 China; Veterinary Medicine Institute, Guangdong Academy of Agricultural Sciences, Guangzhou, 510640 China; Northwestern University, Evanston, IL USA

**Keywords:** Highly pathogenic, Porcine reproductive and respiratory syndrome virus, Real-time RT-PCR

## Abstract

**Background:**

Porcine reproductive and respiratory syndrome virus (PRRSV), and particularly its highly pathogenic genotype (HP-PRRSV), have caused massive economic losses to the global swine industry.

**Results:**

To rapidly identify HP-PRRSV, we developed a direct real-time reverse transcription polymerase chain reaction method (dRT-PCR) that could detect the virus from serum specimen without the need of RNA purification. Our dRT-PCR assay can be completed in 1.5 h from when a sample is received to obtaining a result. Additionally, the sensitivity of dRT-PCR matched that of conventional reverse transcription PCR (cRT-PCR) that used purified RNA. The lowest detection limit of HP-PRRSV was 6.3 TCID_50_ using dRT-PCR. We applied dRT-PCR assay to 144 field samples and the results showed strong consistency with those obtained by cRT-PCR. Moreover, the dRT-PCR method was able to tolerate 5-20% (v/v) serum.

**Conclusions:**

Our dRT-PCR assay allows for easier, faster, more cost-effective and higher throughput detection of HP-PRRSV compared with cRT-PCR methods. To the best of our knowledge, this is the first report to describe a real-time RT-PCR assay capable of detecting PRRSV in crude serum samples without the requirement for purifying RNA. We believe our approach has a great potential for application to other RNA viruses.

**Electronic supplementary material:**

The online version of this article (doi:10.1186/2049-1891-5-45) contains supplementary material, which is available to authorized users.

## Background

Porcine reproductive and respiratory syndrome virus (PRRSV) is a pathogen responsible for significant economic losses to the global swine industry in recent years. The virus causes reproductive failure in pregnant sows and respiratory tract illness in young pigs [1, 2]. PRRSV is a single-stranded 15-kb RNA virus of the family *Arteriviridae* [3]. It can be subdivided into types 1 (European genotype) [2] and 2 (Northern American genotype) [4, 5]. In 2006, a high fever syndrome in swine due to a highly pathogenic type 2 PRRSV (HP-PRRSV) broke out in China [6]. HP-PRRSV infected more than 2 million pigs in 2006 with an average fatality rate of 25%.

The current methods for detecting PRRSV include virus isolation [7], immunoperoxidase monolayer assay (IPMA) [8], and enzyme-linked immunosorbent assay (ELISA) [9, 10]. However, these methods are not specific enough to discriminate between HP-PRRSV and classical strains (C-PRRSV). HP-PRRSV has a discontinuous deletion of 30 amino acids in its non-structural protein 2 (NSP2) [6]. Discrimination of HP-PRRSV from C-PRRSV can be achieved by reverse transcription polymerase chain reaction (RT-PCR) assays targeting differences in the *nsp2* gene [11–15]. However, current PCR methods rely on purification of RNA from a sample, which is time-consuming; if such a step was eliminated, the speed of viral detection could be greatly enhanced.

We sought to develop a direct real-time RT-PCR (dRT-PCR) assay for HP-PRRSV detection in crude samples without subjecting them to RNA extraction and purification procedures. We believe that such a method would greatly simplify and accelerate high throughput viral analysis, along with reducing associated costs and chances of cross contamination.

## Materials and methods

### Viruses, cells and clinical specimens

Classical PRRSV strain CH-1a (GenBank:AY032626) was kindly provided by Dr. Hanzhong Wang (Wuhan Institute of Virology, Chinese Academy of Sciences, Wuhan, China). The highly pathogenic type 2 PRRSV strain 07HBEZ was isolated in 2007 (GenBank:FJ495082.2). Other viruses, including classical swine fever virus (CSFV), pseudorabies virus (PRV), porcine circovirus type 2 (PCV2), porcine parvovirus (PPV) and rotavirus (RV) were stored in our laboratory and used to confirm the specificity of dRT-PCR assay we developed. MARC-145 cells were cultured and maintained in Dulbeco’s modified Eagle’s medium (DMEM) supplemented with 10% newborn calf serum (Gibco) at 37°C, 5% CO_2_. We collected 144 porcine serum samples from more than 10 pig farms across Hubei Province, China from July to September 2012. Briefly, blood samples were taken from the anterior vena cava and placed in 5-mL centrifuge tubes lacking any coagulant or anticoagulant. Samples were centrifuged at 1,000 × *g* for 10 min and the serum was stored at −80°C until required.

Sample collection of porcine sera complied with the regulation of the Ministry of Agriculture of China. Our study was carried out in strict accordance with the recommendations in the Guide for the Care and Use of Laboratory Animals of the Ministry of Agriculture of China. Our study was approved by the Committee on the Ethics of Animal Experiments of Shenzhen University, China (Permit Number: 0156-05/13).

### Primers and probe

HP-PRRSV has a discontinuous deletion of 30 amino acids in the *nsp2* gene. Based on the alignment of *nsp2* sequences published in GenBank, primers were designed using the Primer Premier 5.0. A Taqman probe spanning the flanking sequence of the deleted region of the *nsp2* gene was designed. The primer and probe sequences used in our study were summarized in Table 1.

**Table 1:** **Primer and probe sequences used in our study**

Name	Sequence 5′→3′	Location (GenBank no.)	Product
dHP-PRRSV-F	GGGTCGGCACCAGTTCC	1555-1571 (FJ495082.2)	73 bp
dHP-PRRSV-R	AATCCAGAGGCTCATCCTGGT	1607-1627 (FJ495082.2)	
dHP-PRRSV-pro	FAM-CACCGCGTATAACTGTGACAACAACGC-BHQ1	1574-1600 (FJ495082.2)	

### Direct real-time RT-PCR

We used the Direct One-Step S/P qRT-PCR Taqprobe Kit (VitaNavi Technology, Manchester, USA) to conduct our dRT-PCR assay. This kit contained an S/P RT-PCR enzyme mix comprising reverse transcriptase and highly resistant Taq DNA polymerase, and a 2× S/P RT-PCR master mix containing a PCR enhancer cocktail (PEC)). A volume of each sample (1–5 μL of serum per 25 μL reaction) was directly added to the master reagents that were supplemented with the designed primers and probe. Viral RNA release, reverse transcription (RT) and PCR were all carried out in the same reaction tube. Reactions were optimized by altering the concentration of primers (0.2 ~ 0.4 μmol/L), probe (0.2 ~ 0.4 μmol/L), and enzyme mix (0.75-1.25 μL per 25 μL reaction); and by examining RNA release/RT temperature (55 ~ 60°C), annealing/extension temperature (60 ~ 65°C), annealing/extension time (30 ~ 120 s) and the number of PCR cycles (35 ~ 45 cycles). As a positive control, we used purified RNA from a 100 μL sample of HP-PRRSV-containing serum. This sample had been subjected to RNA extraction using the TaKaRa MiniBEST Viral RNA/DNA Extraction Kit Ver. 4.0 (TaKaRa, Dalian, China) according to the manufacturer’s instructions. The RNA was eluted into 100 μL of RNase-free water, restoring the original specimen volume. PRRSV-negative serum was used as a negative control. Reactions were performed in triplicate and carried out on a StepOne Plus thermocycler (Applied Biosystems). The threshold was set at 0.01 and the threshold cycle (Ct) value was analyzed. Amplification products were confirmed by electrophoresis and DNA sequencing.

### Conventional RT-PCR assay

We used conventional RT-PCR (cRT-PCR) assay employing purified RNA sample as previously described [15].

### Sensitivity of dRT-PCR assay

HP-PRRSV-infected cell supernatants with viral titers of 10^4.8^ TCID_50_/μL was 10-fold serially diluted with PRRSV-negative serum. A 1 μL aliquot of each diluted serum sample was assayed by dRT-PCR in a 25 μL reaction volume. Each reaction was performed in triplicate. The correlation between the titer of HP-PRRSV and Ct value was analyzed.

### Specificity of dRT-PCR assay

The specificity of the dRT-PCR assay was identified by analyzing seven different viruses (HP-PRRSV, C-PRRSV, CSFV, PRV, PCV2, PPV and RV).

### Serum tolerance of dRT-PCR assay

A 1 μL of HP-PRRSV-positive serum was diluted with PRRSV-negative serum to acquire different concentrations of 5, 10, 15, 20, and 25% (v/v) serum in a 25 μL reaction, respectively. Then, all concentrations of sera and a 1 μL of HP-PRRSV RNA were analyzed in parallel in dRT-PCR assays. A 100 μL of HP-PRRSV-positive serum sample was subjected to RNA extraction. The RNA was eluted into 100 μL of RNase-free water, restoring the original specimen volume. Ct values were analyzed and PCR products were evaluated by electrophoresis. Tolerance test was also performed by detecting the same amount of purified RNA added with no serum or with negative serum in various concentrations (5-25%, v/v).

### Statistical analyses

Statistical analyses were performed with GraphPad Prism 5. Correlation coefficient (R^2^) was calculated by linear regression analysis. Repeatability and reproducibility of the dRT-PCR assay was determined by analyzing the mean values and standard deviations of Ct values.

## Results

### dRT-PCR assay

Optimized dRT-PCR assay (25 μL) contained 12.5 μL 2× S/P RT-PCR master mix, 1 μL S/P RT-PCR enzyme mix, 0.4 μmol/L forward primer, 0.4 μmol/L reverse primer, 0.2 μmol/L probe, template (1–5 μL) and water. Reactions were conducted at 55°C for 30 min (for viral RNA release and RT), then 95°C for 5 min, followed by 40 cycles of 94°C for 20 s and 60°C for 40 s.

### Sensitivity of dRT-PCR assay

**Figure 1:**
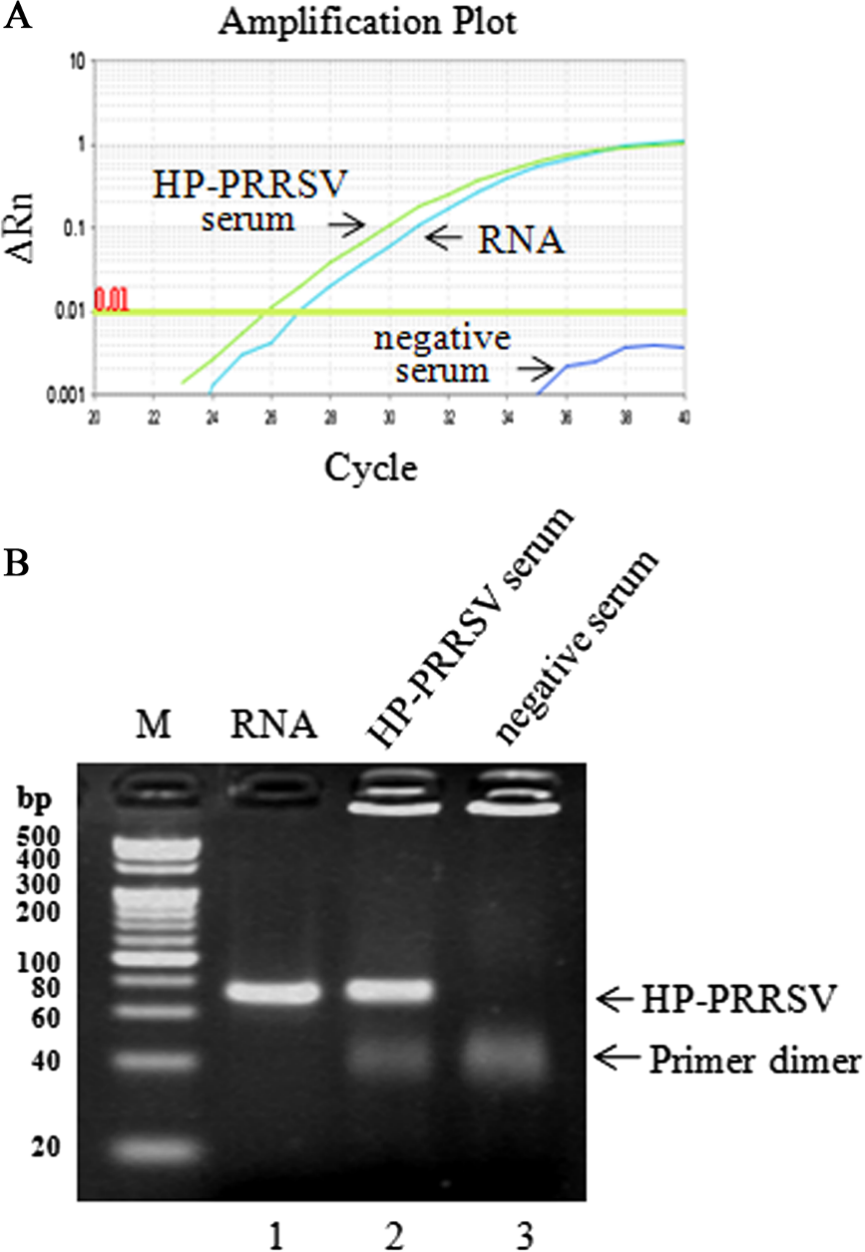
**Use of a dRT-PCR assay to efficiently detect HP-PRRSV in crude serum samples. (A)** Amplification plots for dRT-PCR and cRT-PCR assays. Threshold was set at 0.01. **(B)** Amplicons from the dRT-PCR assays and controls were electrophoresed on 3.5% (w/v) agarose gels. The arrows indicate the 73-bp target bands for HP-PRRSV. A 1 μL of purified HP-PRRSV RNA (Lane 1), 1 μL of HP-PRRSV-containing serum (Lane 2) and 1 μL of PRRSV-negative serum (Lane 3) were added to a 25 μL dRT-PCR mixture, respectively. All assays were performed in triplicate.

**Figure 2:**
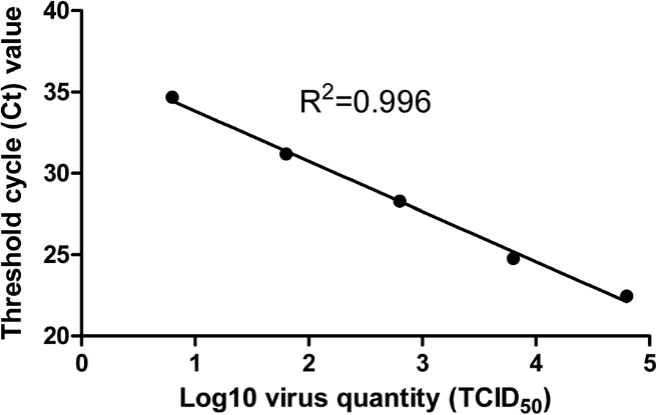
**Sensitivity of the dRT-PCR assay.** The HP-PRRSV-infected cell supernatant was 10-fold serially diluted with PRRSV-negative serum. Viral titers ranged from 10^4.8^ TCID_50_/μL to 10^0.8^ TCID_50_/μL. Each diluted sample was assayed in triplicate.

The sensitivity of dRT-PCR was compared to that of conventional RT-PCR (cRT-PCR) using purified RNA. When using 1 μL HP-PRRSV serum sample, the average Ct value produced by dRT-PCR was 25.91, while that for cRT-PCR was 26.93 (Figure 1), indicating the sensitivity of dRT-PCR was even better than that of cRT-PCR. We further tested the sensitivity using HP-PRRSV-infected cell supernatants of known viral titer. The correlation coefficient (R^2^) for virus input and Ct values was 0.996 when viral titer ranged from 10^4.8^ TCID_50_/μL to 10^0.8^ TCID_50_/μL (Figure 2). The lowest detection limit of HP-PRRSV was 6.3 TCID_50_.

### Specificity and accuracy of dRT-PCR assay

The Taqman probe we designed only worked when the 30-amino acid coding sequence of *nsp2* gene was deleted, allowing for specific discrimination of HP-PRRSV from C-PRRSV and other viruses (CSFV, PRV, PCV2, PPV and RV; Additional file 1). To further evaluate the accuracy of dRT-PCR assay, we analyzed a profusion of field serum samples. These samples had been previously evaluated using a cRT-PCR method developed by Yang [15]. A double-blind analysis was performed for the dRT-PCR assay. Of the 144 samples assayed, 94 (65.3%) were HP-PRRSV positive while 50 (34.7%) were negative, showing strong consistency with that obtained by cRT-PCR method (Table 2).

**Table 2:** **Application of dRT-PCR and cRT-PCR assays on field serum samples**

Method	No. of specismen	HP-PRRSV positive	HP-PRRSV negative	Positive rate, %	Correlation, %
dRT-PCR	144	94	50	65.3	100
cRT-PCR^a^	144^b^	94	50	65.3	100

^a^The cRT-PCR data were provided by Yang [15].

^b^These samples had also been detected using immunochromatochemistry by Yang [15].

### Repeatability and reproducibility of dRT-PCR

**Figure 3:**
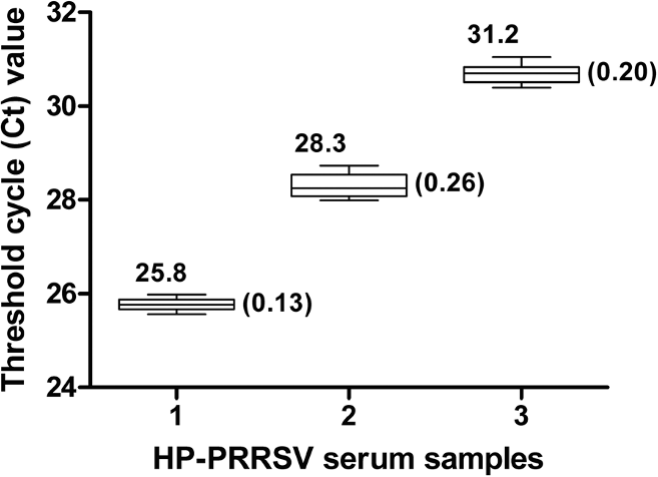
**Repeatability and reproducibility of the dRT-PCR assay.** Three field serum samples with different virus titers were selected and assayed. Mean values and standard deviations of Ct values are depicted above and beside the box plots, respectively.

To test the repeatability and reproducibility of the dRT-PCR, three serum samples with differing viral titers were selected. Each sample was assayed six times, and these experiments were repeated three times on different days; with a total of 18 replicates available for analysis (Figure 3 and Additional file 2). The standard deviations of Ct values ranged from 0.13 to 0.26, indicating a good repeatability and reproducibility of the method.

### Inhibitor tolerance of dRT-PCR assay

**Figure 4:**
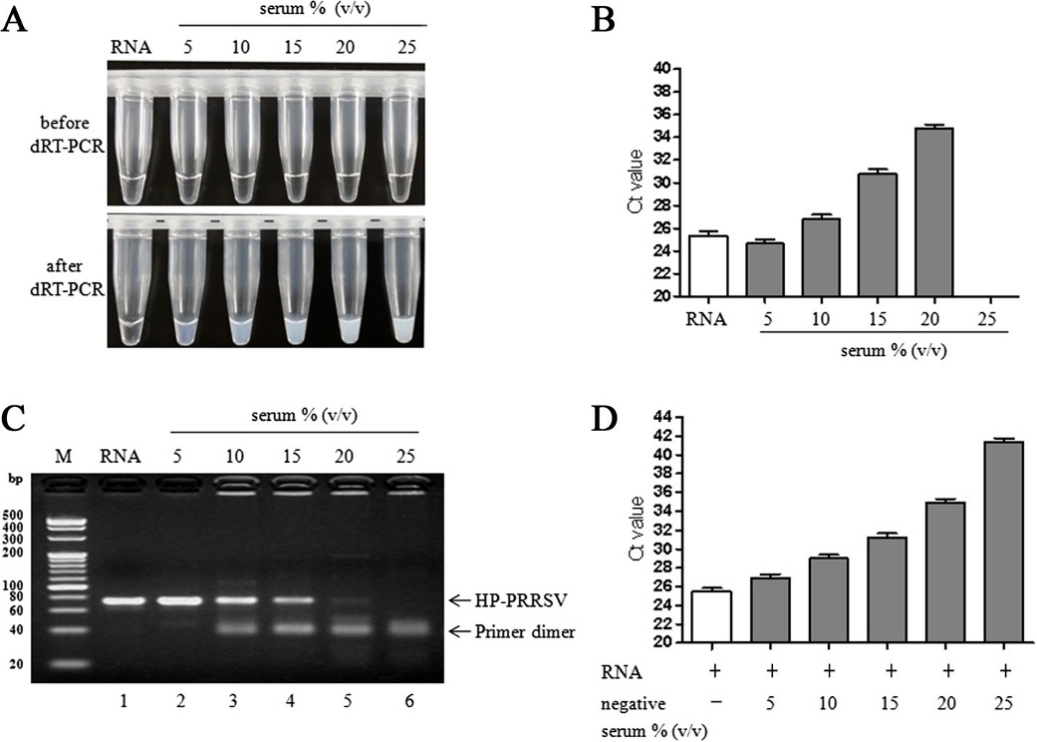
**Serum tolerance of the dRT-PCR assay. (A)** The dRT-PCR mixtures became turbid following the completion of thermal cycling. **(B)** Ct values of dRT-PCR assay where different concentration of HP-PRRSV serum and purified RNA were used. **(C)** Amplicons of dRT-PCR assays were electrophoresed on 3.5% (w/v) agarose gel. Lane 1: 1 μL of purified HP-PRRSV RNA derived from 1 μL of HP-PRRSV-positive serum; Lane 2–6: 1 μL of HP-PRRSV-positive serum diluted with negative serum to acquire different serum concentrations of 5, 10, 15, 20, and 25% (v/v) in a 25 μL reaction, respectively. The arrow indicates the 73-bp target bands for HP-PRRSV. **(D)** Ct values of dRT-PCR assays detecting the same amount of purified RNA supplemented with no serum or with negative serum in various concentrations (5-25%, v/v).

To determine to what extent this method could endure inhibitors, we performed dRT-PCR assay using a wide range of serum concentrations (5-25%, v/v). Reaction mixture adding with purified RNA was transparent after the reaction was completed while the reaction mixtures using crude serum samples were no longer transparent at the end of the reactions (Figure 4A). They became increasingly turbid as the serum concentration increased. Interestingly, the dRT-PCR assay remained functional when the serum concentration was in the range 5–20% as there were amplification plots (Ct value < 35), and the target PCR band could be observed in electrophoresis (Figure 4B and C). The most robust signals were detected when the serum concentration was between 5% and 10%.

We also tested the tolerance of dRT-PCR in a way that used the same amount of purified RNA added with no serum or with negative serum in various concentrations (5-25%, v/v). The results showed that significant signals were obtained among 5-10% serum although the reactions were gradually inhibited as serum concentrations increased (Figure 4D). It also suggested that notable RNA loss might have occured during RNA purification process whereas dRT-PCR assay could effectively avoid this problem.

## Discussion

Development of a rapid and high-throughput RT-PCR method for monitoring HP-PRRSV infections could be significant for quickly sensing and responding to epidemics. One strategy to shorten detection time would be to eliminate the time-consuming RNA purification process. However, abundant PCR inhibitors exist in crude biological samples, such as IgG, hemoglobin, hemin and lactoferrin in blood or serum/plasma [16, 17], and humic acid in soil [18, 19]. The presence of these inhibitors suppresses the activity of DNA polymerase, necessitating a nucleic acid purification prior to conducting any type of PCR assay.

In 2009, Barnes reported an N-terminal 278-aa truncated Taq DNA polymerase Klentaq1 that could amplify genomic DNA in the presence of 5-10% whole blood [20]. This seemed unlikely given that the full-length Taq DNA polymerase enzyme can be inhibited with 0.1 ~ 1% blood; however, this finding inspired researchers to screen for more enzyme mutants with higher resistance. Further studies showed that amino acids relating to high resistance were located at codons 706–708. Subsequently, all 19 possible amino-acid variants of Taq DNA polymerase at codon 708 were tested. Two mutant alternatives, KT 10 (E708K) and Taq 22 (E708Q) were revealed to exhibit higher tolerance and remained functional even in 20% blood, and when humic acid (0.2–0.4 μg/mL) and lactoferrin (2.6–5.2 mmol/L) were present.

Betaine is a commonly used PCR enhancer capable of reducing melting temperature and inhibiting the formation of secondary structure in templates [21, 22]. L-carnitine and D-(+)-trehalose function in similar ways to betaine [23]. The nonionic detergent NP-40 can enhance DNA sequencing by lowering the number of non-specific bands [24]. A PEC of L-carnitine, D-(+)-trehalose and NP-40 can help in the amplification of GC-rich templates and boost reactions rich with inhibitors [23]. This PEC is included in the 2× S/P RT-PCR master mix we used for our dRT-PCR assay to overcome the various inhibitors in serum.

Real-time fluorescence PCR using crude blood or serum/plasma samples is more challenging than PCR methods that use purified nucleic acids as the fluorescent signal is quenched by compounds such as hemoglobin and heme. There were studies reporting direct RT-PCR assays without RNA purification for the detection of Norovirus and bovine viral diarrhoea virus [25, 26]. Nonetheless, none of them were real-time PCR assay. African Chikungunya virus could be detected by TaqMan RT-PCR assay without RNA extraction with less than 1% serum [27]. However, our dRT-PCR assay remains functional in the presence of rather high concentrations of serum because of the previously described tolerant Taq DNA polymerase and PEC. To the best of our knowledge, this is the first successful real-time RT-PCR assay for HP-PRRSV using a crude specimen where nucleic acid extraction and purification has not been conducted.

Another factor ensuring success in our dRT-PCR assay is the efficient release of viral RNA and the subsequent RT reaction. In conventional RT-PCR, chemicals such as TRIzol help denature the capsid protein and release the viral RNA. A high temperature also facilitates release of viral RNA. As an example, pretreatment at 85°C for 1 min was used to release noroviruses RNA [25]; Treatment at 95°C for 4 min was used to liberate of bovine viral diarrhoea virus RNA [26]. However, pretreatment at high temperatures will result in degradation of RNA. In our experiment, we adopted a simple one-step procedure for RNA release and RT. Treating samples at 55°C for 30 min was efficient for both RNA release and subsequent RT for HP-PRRSV. The detergent NP-40 also promotes denaturation of viral capsid protein. Other compounds in the PEC also improve the resistance of reverse transcriptase to inhibitors in serum [23].

As shown in Figures 1 and 4, the sensitivity of the dRT-PCR assay was comparable to, and in some cases, exceeded that of conventional RT-PCR assay. These results suggested that remarkable RNA loss might frequently occurr during RNA purification step in conventional RT-PCR assay, while dRT-PCR assay could effectively conquer this problem.

Given that real-time RT-PCR can be performed in 96- and 384-well formats currently, thousands of field samples can be assayed within a single day when the need of RNA purification is eliminated, thus our dRT-PCR assay providing a high-throughput detection for HP-PRRSV. It is of great significance during an epidemic outbreak.

## Conclusions

We developed a direct real-time RT-PCR assay to determine the presence of HP-PRRSV in crude serum sample. Our assay allowed for more rapid and higher throughput detection of the virus. This approach has a great potential for application to other RNA viruses.

## Electronic supplementary material

Additional file 1: **Specificity of the** **dRT-PCR** **assay.** Primers and probe based on the 30 amino-acid deletion in the HP-PRRSV *nsp2* gene were used in dRT-PCR assays for seven different viruses. Except for HP-PRRSV, there was no significant amplification signal for six other viruses (C-PRRSV, CSFV, PRV, PCV2, PPV and RV), indicating high specificity of the dRT-PCR. (DOCX 368 KB)

Additional file 2: **Ct values for** **HP-PRRSV** **in repeatability and** **reproducibility assays using our developed** **dRT-PCR** **method.** (DOCX 14 KB)

## References

[1] Rossow KD (1998). Porcine reproductive and respiratory syndrome. Vet Pathol.

[2] Albina E (1997). Epidemiology of porcine reproductive and respiratory syndrome (PRRS): an overview. Vet Microbiol.

[3] Cavanagh D (1997). Nidovirales: a new order comprising Coronaviridae and Arteriviridae. Arch Virol.

[4] Elazhary Y, Weber J, Bikour H, Morin M, Girard C (1991). ‘Mystery swine disease’ in Canada. Vet Rec.

[5] Wensvoort G, Terpstra C, Pol JM, ter Laak EA, Bloemraad M, de Kluyver EP, Kragten C, van Buiten L, den Besten A, Wagenaar F (1991). Mystery swine disease in The Netherlands: the isolation of Lelystad virus. Vet Q.

[6] Tian K, Yu X, Zhao T, Feng Y, Cao Z, Wang C, Hu Y, Chen X, Hu D, Tian X, Liu D, Zhang S, Deng X, Ding Y, Yang L, Zhang Y, Xiao H, Qiao M, Wang B, Hou L, Wang X, Yang X, Kang L, Sun M, Jin P, Wang S, Kitamura Y, Yan J, Gao GF (2007). Emergence of fatal PRRSV variants: unparalleled outbreaks of atypical PRRS in China and molecular dissection of the unique hallmark. PLoS One.

[7] Mengeling WL, Lager KM, Wesley RD, Clouser DF, Vorwald AC, Roof MB (1999). Diagnostic implications of concurrent inoculation with attenuated and virulent strains of porcine reproductive and respiratory syndrome virus. Am J Vet Res.

[8] Nodelijk G, Wensvoort G, Kroese B, van Leengoed L, Colijn E, Verheijden J (1996). Comparison of a commercial ELISA and an immunoperoxidase monolayer assay to detect antibodies directed against porcine respiratory and reproductive syndrome virus. Vet Microbiol.

[9] Albina E, Leforban Y, Baron T, Plana Duran JP, Vannier P (1992). An enzyme linked immunosorbent assay (ELISA) for the detection of antibodies to the porcine reproductive and respiratory syndrome (PRRS) virus. Ann Rech Vet.

[10] Dea S, Wilson L, Therrien D, Cornaglia E (2001). Detection of antibodies to the nucleocapsid protein of PRRS virus by a competitive ELISA. Adv Exp Med Biol.

[11] Chai Z, Ma W, Fu F, Lang Y, Wang W, Tong G, Liu Q, Cai X, Li X (2013). A SYBR Green-based real-time RT-PCR assay for simple and rapid detection and differentiation of highly pathogenic and classical type 2 porcine reproductive and respiratory syndrome virus circulating in China. Arch Virol.

[12] Wernike K, Hoffmann B, Dauber M, Lange E, Schirrmeier H, Beer M (2012). Detection and typing of highly pathogenic porcine reproductive and respiratory syndrome virus by multiplex real-time rt-PCR. PLoS One.

[13] Chen NH, Chen XZ, Hu DM, Yu XL, Wang LL, Han W, Wu JJ, Cao Z, Wang CB, Zhang Q, Wang BY, Tian KG (2009). Rapid differential detection of classical and highly pathogenic North American Porcine Reproductive and Respiratory Syndrome virus in China by a duplex real-time RT-PCR. J Virol Methods.

[14] Xiao XL, Wu H, Yu YG, Cheng BZ, Yang XQ, Chen G, Liu DM, Li XF (2008). Rapid detection of a highly virulent Chinese-type isolate of Porcine Reproductive and Respiratory Syndrome Virus by real-time reverse transcriptase PCR. J Virol Methods.

[15] Yang K, Li Y, Duan Z, Guo R, Liu Z, Zhou D, Yuan F, Tian Y (2013). A one-step RT-PCR assay to detect and discriminate porcine reproductive and respiratory syndrome viruses in clinical specimens. Gene.

[16] Queipo-Ortuno MI, De Dios Colmenero J, Macias M, Bravo MJ, Morata P (2008). Preparation of bacterial DNA template by boiling and effect of immunoglobulin G as an inhibitor in real-time PCR for serum samples from patients with brucellosis. Clin Vaccine Immunol.

[17] Al-Soud WA, Radstrom P (2001). Purification and characterization of PCR-inhibitory components in blood cells. J Clin Microbiol.

[18] Tsai YL, Olson BH (1992). Rapid method for separation of bacterial DNA from humic substances in sediments for polymerase chain reaction. Appl Environ Microbiol.

[19] Watson RJ, Blackwell B (2000). Purification and characterization of a common soil component which inhibits the polymerase chain reaction. Can J Microbiol.

[20] Kermekchiev MB, Kirilova LI, Vail EE, Barnes WM (2009). Mutants of Taq DNA polymerase resistant to PCR inhibitors allow DNA amplification from whole blood and crude soil samples. Nucleic Acids Res.

[21] Henke W, Herdel K, Jung K, Schnorr D, Loening SA (1997). Betaine improves the PCR amplification of GC-rich DNA sequences. Nucleic Acids Res.

[22] Musso M, Bocciardi R, Parodi S, Ravazzolo R, Ceccherini I (2006). Betaine, dimethyl sulfoxide, and 7-deaza-dGTP, a powerful mixture for amplification of GC-rich DNA sequences. J Mol Diagn.

[23] Zhang Z, Kermekchiev MB, Barnes WM (2010). Direct DNA amplification from crude clinical samples using a PCR enhancer cocktail and novel mutants of Taq. J Mol Diagn.

[24] Petry H, Bachmann B, Luke W, Hunsmann G (1996). PCR sequencing with the aid of detergents. Methods Mol Biol.

[25] Nishimura N, Nakayama H, Yoshizumi S, Miyoshi M, Tonoike H, Shirasaki Y, Kojima K, Ishida S (2010). Detection of noroviruses in fecal specimens by direct RT-PCR without RNA purification. J Virol Methods.

[26] Bachofen C, Willoughby K, Zadoks R, Burr P, Mellor D, Russell GC (2013). Direct RT-PCR from serum enables fast and cost-effective phylogenetic analysis of bovine viral diarrhoea virus. J Virol Methods.

[27] Pastorino B, Bessaud M, Grandadam M, Murri S, Tolou HJ, Peyrefitte CN (2005). Development of a TaqMan® RT-PCR assay without RNA extraction step for the detection and quantification of African Chikungunya viruses. J Virol Methods.

