# Supplementary material for: A direct real-time polymerase chain reaction assay for rapid high-throughput detection of highly pathogenic North American porcine reproductive and respiratory syndrome virus in China without RNA purification
Source: J Anim Sci Biotechnol. 2014 Oct 2;5(1):45. doi: 10.1186/2049-1891-5-45 (PMC4198619; doi:10.1186/2049-1891-5-45)
Supplement: Supplementary file 1 — Additional file 1: Specificity of the dRT-PCR assay. Primers and probe based on the 30 amino-acid deletion in the HP-PRRSV nsp2 gene were used in dRT-PCR assays for seven different viruses. Except for HP-PRRSV, there was no significant amplification signal for six other viruses (C-PRRSV, CSFV, PRV, PCV2, PPV and RV), indicating high specificity of the dRT-PCR. (DOCX 368 KB) [file 40104_2014_126_MOESM1_ESM.docx]

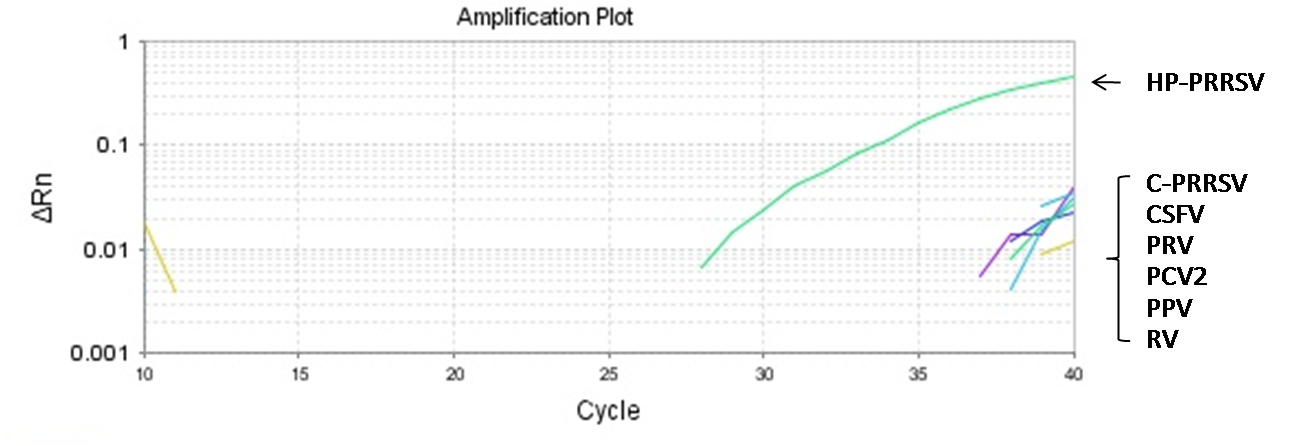


Additional file 1 **Specificity of dRT-PCR**. The primers and probe that based on the 30 amino-acid delection in HP-PRRSV *nsp2* gene were used for dRT-PCR assays of seven different viruses. Except for HP-PRRSV, there was no significant amplification signal for other six viruses, including C-PRRSV, CSFV, PRV, PCV2, PPV and RV, indicating a good specificity of dRT-PCR.
