# Supplementary material for: A direct real-time polymerase chain reaction assay for rapid high-throughput detection of highly pathogenic North American porcine reproductive and respiratory syndrome virus in China without RNA purification
Source: J Anim Sci Biotechnol. 2014 Oct 2;5(1):45. doi: 10.1186/2049-1891-5-45 (PMC4198619; doi:10.1186/2049-1891-5-45)
Supplement: Supplementary file 2 — Additional file 2: Ct values for HP-PRRSV in repeatability and reproducibility assays using our developed dRT-PCR method. (DOCX 14 KB) [file 40104_2014_126_MOESM2_ESM.docx]

Additional file 2

The Ct values of HP-PRRSV in repeatability and reproducibility assay using dRT-PCR

| Replicate  number | Ct value | | |
| --- | --- | --- | --- |
|  | Sample 1 | Sample 2 | Sample 3 |
| 1 | 25.65 | 28.01 | 30.97 |
| 2 | 25.79 | 28.28 | 31.24 |
| 3 | 25.66 | 28.03 | 30.99 |
| 4 | 25.89 | 28.60 | 31.43 |
| 5 | 25.99 | 28.77 | 31.54 |
| 6 | 25.76 | 28.19 | 31.17 |
| 7 | 25.79 | 28.29 | 31.29 |
| 8 | 25.88 | 28.55 | 31.39 |
| 9 | 25.67 | 28.10 | 31.05 |
| 10 | 25.85 | 28.47 | 31.35 |
| 11 | 25.63 | 28.21 | 31.20 |
| 12 | 25.70 | 28.16 | 31.12 |
| 13 | 25.88 | 28.36 | 31.31 |
| 14 | 26.03 | 28.81 | 31.54 |
| 15 | 25.77 | 28.25 | 31.23 |
| 16 | 25.54 | 27.95 | 30.90 |
| 17 | 25.62 | 27.99 | 30.95 |
| 18 | 25.69 | 28.13 | 31.09 |
|  |  |  |  |
| mean ±  standard deviations | 25.77 ± 0.13 | 28.29 ± 0.26 | 31.21 ± 0.20 |
